# De novo transcriptome analysis of the critically endangered alpine Himalayan herb *Nardostachys jatamansi* reveals the biosynthesis pathway genes of tissue-specific secondary metabolites

**DOI:** 10.1038/s41598-020-74049-1

**Published:** 2020-10-14

**Authors:** Nisha Dhiman, Anil Kumar, Dinesh Kumar, Amita Bhattacharya

**Affiliations:** 1 Division of Biotechnology, CSIR-Institute of Himalayan Bioresource Technology, Palampur, H.P. 176061 India; 2 Academy of Scientific and Innovative Research (AcSIR), Ghaziabad, 201002 India; 3 Chemical Technology Division, CSIR-Institute of Himalayan Bioresource Technology, Palampur, H.P. 176061 India

**Keywords:** Biotechnology, Molecular biology, Plant sciences

## Abstract

The study is the first report on de novo transcriptome analysis of *Nardostachys jatamansi*, a critically endangered medicinal plant of alpine Himalayas. Illumina GAIIx sequencing of plants collected during end of vegetative growth (August) yielded 48,411 unigenes. 74.45% of these were annotated using UNIPROT. GO enrichment analysis, KEGG pathways and PPI network indicated simultaneous utilization of leaf photosynthates for flowering, rhizome fortification, stress response and tissue-specific secondary metabolites biosynthesis. Among the secondary metabolite biosynthesis genes, terpenoids were predominant. UPLC-PDA analysis of in vitro plants revealed temperature-dependent, tissue-specific differential distribution of various phenolics. Thus, as compared to 25 °C, the phenolic contents of both leaves (gallic acid and rutin) and roots (*p*-coumaric acid and cinnamic acid) were higher at 15 °C. These phenolics accounted for the therapeutic properties reported in the plant. In qRT-PCR of in vitro plants, secondary metabolite biosynthesis pathway genes showed higher expression at 15 °C and 14 h/10 h photoperiod (conditions representing end of vegetative growth period). This provided cues for in vitro modulation of identified secondary metabolites. Such modulation of secondary metabolites in in vitro systems can eliminate the need for uprooting *N. jatamansi* from wild. Hence, the study is a step towards effective conservation of the plant.

## Introduction

*Nardostachys jatamansi* (D.Don) DC. of family Caprifoliaceae is an important but critically endangered medicinal herb inhabiting specific ecological niches of mostly inaccessible locales of alpine and sub-alpine Himalayas (2200 to over 4800 m amsl at 40–70° incline). The plant propagates vegetatively through rhizomes in nature^1,2^. The rhizome not only supports flowering and sexual reproduction at the end of growth period but also readies the plant for opportunistic growth, immediately, after the return of favourable season. Importantly, it helps the plant persist in its specific ecological niche of alpine habitats. Aerial parts comprised of a rosette of elongated leaves emerge directly from the rhizomes every year during favourable seasons to subsequently form a dense clump of mature clonal plants. Flower bearing shoots emerge from rhizomes and set seeds during late August (month representing the end of favourable season). After flowering and wind dispersal of seeds to new locations during September, the aerial parts of the plant undergo senescence but the perennating rhizome continues to grow and fortify itself. Even during this time, new shoot bud meristems develop from the rhizomes as preparedness for fresh vegetative growth in the next or the following season of favourable conditions. Apparently, diverse functions of growth and fortification occur simultaneously in the rhizomes during the end of favourable growth period. Surely, an underlying complex machinery govern all these processes leading to the biosynthesis of an array of valuable metabolites. However, there is no information available on *N. jatamansi* to this effect. Hence, de novo transcriptome analysis of the plant parts at flowering stage was undertaken to understand the genetic machinery governing the various processes in the plant during the end of growth period. The study also aimed at understanding the various metabolic processes occurring in *N. jatamansi* during the end of growth period.

Such information is important for a critically endangered plant like *N. jatamansi*. The plant is uprooted for its roots and rhizomes from wild because of its immense therapeutic use in the Indian, Chinese, Japanese, Tibetan and other traditional systems of medicine^3^. As per National Medicinal Plant Board, Government of India, marketing and trade report, the annual demand for the plant is about 500–1000 metric tons of rhizomes^4^. *N. jatamansi* is also one of the most traded plants in India and figures in the Annexure no. 3 of Appendix II of ‘Convention on International Trade in Endangered Species of Wild Fauna and Flora, (CITES)’, negative list of exports, and also list of unethical uses of rare and threatened plants and animal products. The plant is also categorised as ‘critically endangered A2cd ver 3.1’ in the IUCN red list of threatened plants’. In this regard, the findings of our study are expected to provide information on the biosynthetic pathway genes of the various secondary metabolites present in *N. jatamansi*. The key regulators identified in the study are also envisaged to provide cues for production and modulation of important metabolites in in vitro systems like callus and cell cultures. Such alternative approaches for secondary metabolite production can not only eliminate the need for indiscriminate uprooting of *N. jatamansi* plants from wild but also arrest the dwindling of natural populations.

## Results

### Transcriptome sequencing and de novo sequence assembly

The Illumina GA IIx sequencing platform was used for de novo transcriptome of *N. jatamansi* at flowering stage. After sequencing, 13.92 and 9.53 million raw reads (a total of 23.45 million raw reads) were obtained from leaves and rhizomes, respectively. After filtering out the adapter sequences, the reads of low quality and the reads of short length, a total of 13.87 and 9.27 (23.14) million reads of high quality were obtained from the leaves and rhizomes, respectively (Supplementary Table 1). When these were assembled de novo using Trinity (default k-mers i.e., 25) and clustered using CD-HIT, a total of 35,777 and 32,491 transcripts from leaves and rhizomes, respectively were obtained. Finally, these were assembled de novo into 48,411 unigenes. The average contig length of the reads was 742.6 bp ± 590.4 and 731.2 ± 631.1 and their average N50 value was 1110 and 1101 bp in leaves and rhizomes, respectively. The statistical summary of the data is provided in Supplementary Table 1, and the length distribution of the clustered transcripts in Supplementary Fig. 1.

After similarity search of sequences, a total of 74.45% transcripts were annotated functionally using UNIPROT database. However, a number of unigenes could not be annotated functionally. The e-value distribution of the annotated transcripts is given in Supplementary Fig. 2.

### Functional annotation of unigenes

**Figure 1:**
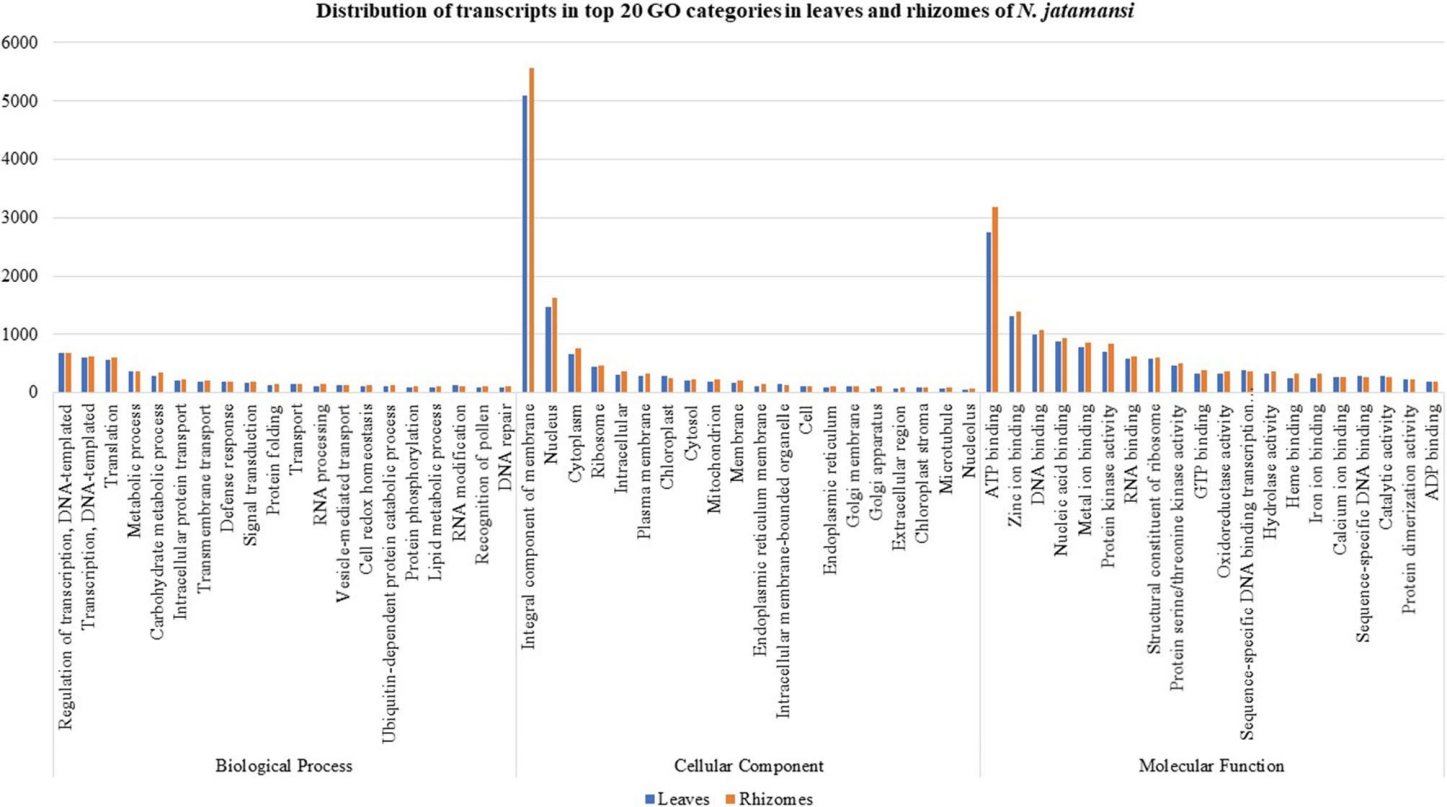
Transcript distribution in top 20 GO categories in *N. jatamansi* leaves and rhizomes under Biological process, Cellular component and Molecular function.

The unigenes obtained above could be categorized into biological process, cellular component and molecular function according to GO analysis. Out of 74.76 and 74.14% transcripts of leaves and rhizomes, respectively, a total of 24,292 and 26,527 were assigned to either one of the GO categories. Of these, 14,116 unigenes of the two tissues were assigned to biological process term (1496), 14,310 unigenes under 440 cellular component term and 26,565 unigenes under 1332 molecular function term. The top 20 classes from each category are represented in Fig. 1. Most of the genes belonged to regulation of transcription, metabolic process, carbohydrate metabolic process, transport related terms, defence response and signal transduction under the ‘biological process’. Under ‘cellular component’, integral component of membrane, nucleus, cytoplasm, ribosome, intracellular and plasma membrane were the predominant categories. Under ‘molecular function’ however, ATP, zinc ion, DNA, nucleic acid and metal ion binding as well as protein kinase activity and RNA binding were amongst the top 10 major classes.

On homology search for species similarity, the transcripts of *N. jatamansi* showed maximum similarity with *Vitis vinifera* followed by *Daucus carota* subsp. *sativus*, *Coffea canephora* and *Cynara cardunculus* var. *scolymus*. The top 20 species having highest similarity with *N. jatamansi* are represented in Supplementary Fig. 3. Interestingly, only 11 transcripts were common to both *N. jatamansi* and *Valeriana officinalis*, the other genera of family Caprifoliaceae.

Among the sequences searched against KEGG pathway database, a total of 4985 and 4971 transcripts were assigned to 137 and 140 pathways in leaves and rhizomes, respectively (Supplementary Table 3). The most abundant pathways in the leaves and rhizomes were ribosome comprising of 258 and 261 genes followed by carbon metabolism (172 genes of each tissue), spliceosome (172 and 181), biosynthesis of amino acids (169 and 171 genes) and protein processing in endoplasmic reticulum (146 and 136 genes). In the top 20 KEGG pathways, significantly higher number of unigenes of ribosome, spliceosome, carbon metabolism, biosynthesis of amino acids, protein processing in endoplasmic reticulum, RNA transport, purine metabolism, plant–pathogen interaction, plant hormone signal transduction and oxidative phosphorylation were recorded (Supplementary Fig. 4). In addition, transcripts of 41 and 43 different primary and secondary metabolite biosynthesis pathways were identified in the leaves and rhizomes, respectively. The distribution of the transcripts in the different metabolic pathways of leaves and rhizomes is depicted in Supplementary Table 3.

### SSR identification

After SSR prediction analysis using MISA perl script, a total of 7240 and 7458 SSRs were recorded in 5800 (22.28%) and 6032 (20.84%) transcripts of leaves and rhizomes, respectively. Based on SSR distribution and mining of transcripts, a total of 1138 and 1123 transcripts having more than one SSR were identified in leaves and rhizomes, respectively. The most abundant SSR repeat types included the mononucleotides (35.73 and 33.66%) followed by dinucleotides (32.59 and 33.45%) in leaves and rhizomes, respectively (Supplementary Fig. 5).

### Abundance analysis of tissue specific differentially expressed genes (DEGs)

The transcripts having log_2_ fold change (FC) value above 2 and less than − 2, and *p*-value below 0.5 in edgeR software were considered (Supplementary Table 4). On analysing the differential expression in pair-wise comparison, 4177 and 4810 transcripts were upregulated in leaves and rhizomes, respectively. Among these however, 1403 from leaves and 1784 from rhizomes could not be annotated functionally. Moreover, 1612 and 1632 were uncharacterized in leaves and rhizomes, respectively (Supplementary Fig. 6).

### GO enrichment

In GO enrichment analysis of the significantly enriched DEGs of leaves and rhizomes (Supplementary Fig. 7a–f), processes related to photosynthesis (GO: 0015979) and related terms (GO:0019684, GO: 0009765), generation of precursor metabolites and energy (GO: 0006091), biosynthetic processes (GO id: 0009058), translation related processes (GO: 0006412, 0019538, 0044267, 0034645, 0006518, 0043604, 0043603, 0043043, 901565), nucleotide triphosphate metabolism related processes (GO ids: 0009141, 0009144, 000936, 0009142, 0009201, 0009145, 0009205, 0009206) and nucleotide biosynthetic processes (GO ids: 0006163, 0042278, 0042451, 0045129, 0046128) were significantly enriched in the leaves.

In rhizomes, the top enriched classes in ‘biological process’ belonged to organonitrogen compound metabolic process (GO: 1901564) and related terms, organonitrogen compound biosynthetic process (GO: 1901566), peptide metabolic process (GO: 0006518), cellular amide metabolic process (GO: 0043603), amide biosynthetic process (GO: 0043604), peptide biosynthetic process (GO: 0043043), cellular carbohydrate metabolic process (GO: 0044275), cellulose catabolic process (GO: 0030245), beta-glucan catabolic process (GO: 0051275), beta-glucan metabolic process (GO: 0051273) and cellular polysaccharide metabolic process (GO: 044247). In addition, peptide biosynthetic process, response to oxidative stress (GO: 006979), cell wall organization or biogenesis, secondary metabolic processes (GO: 0019748), phenylpropanoid and lignin metabolism (GO: 0009698, GO: 0009808) were significantly enriched.

### Identification of transcription factors

**Figure 2:**
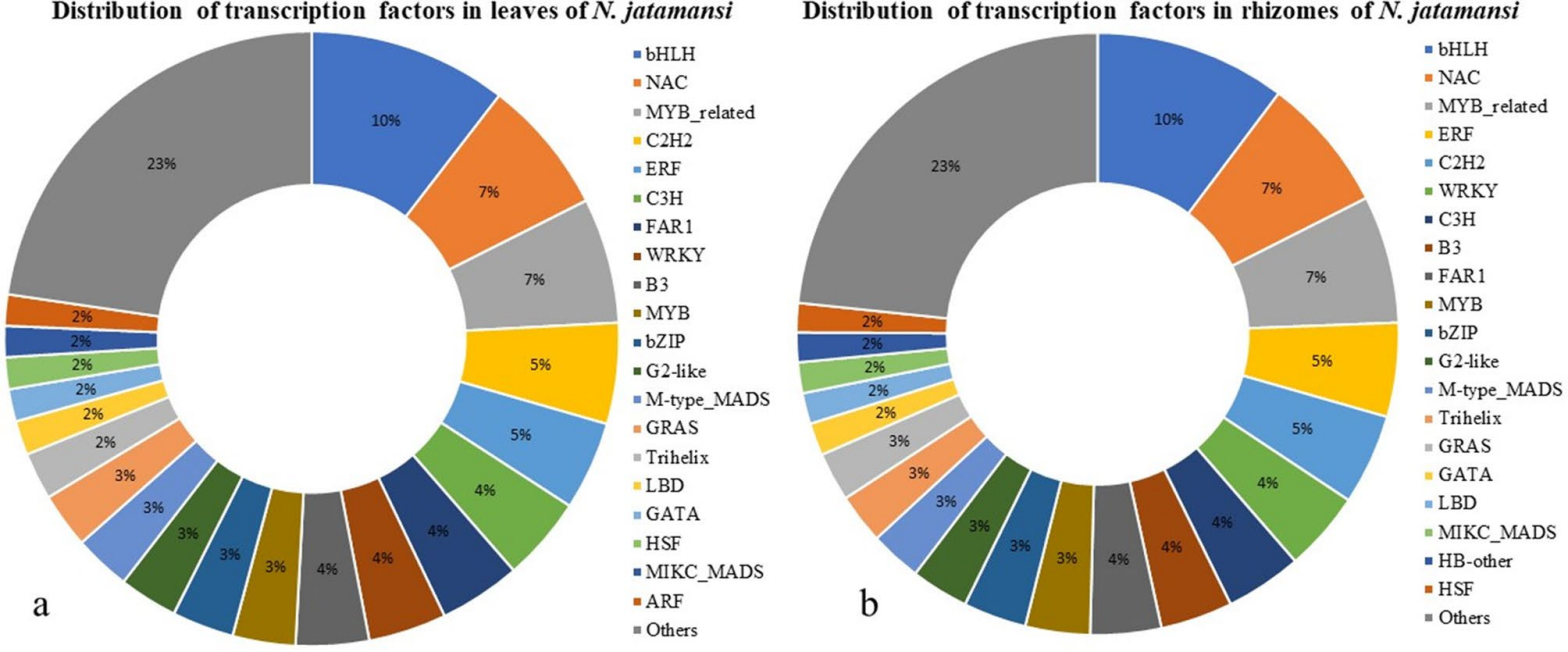
Transcription factor families in (**a**) leaves and (**b**) rhizomes of *N. jatamansi.*

In Plant TFDB Homology search (E value cut off of 10^–5^), a total of 9982 and 10,655 transcripts belonging to 57 TF families were recorded in leaves and rhizomes, respectively. Of these, the bHLH family was the most dominant (10.45 and 10.27%). This was followed by NAC (7.12 and 7.27%), MYB-related (6.59 and 6.86%), ERF (4.68 and 5.04%) and C_2_H_2_ (5.31 and 4.87%) families in leaves and rhizomes, respectively. The top 20 families of transcription factors are represented in Fig. 2 and Supplementary Table 5.

### Protein–protein interaction (PPI) network

**Figure 3:**
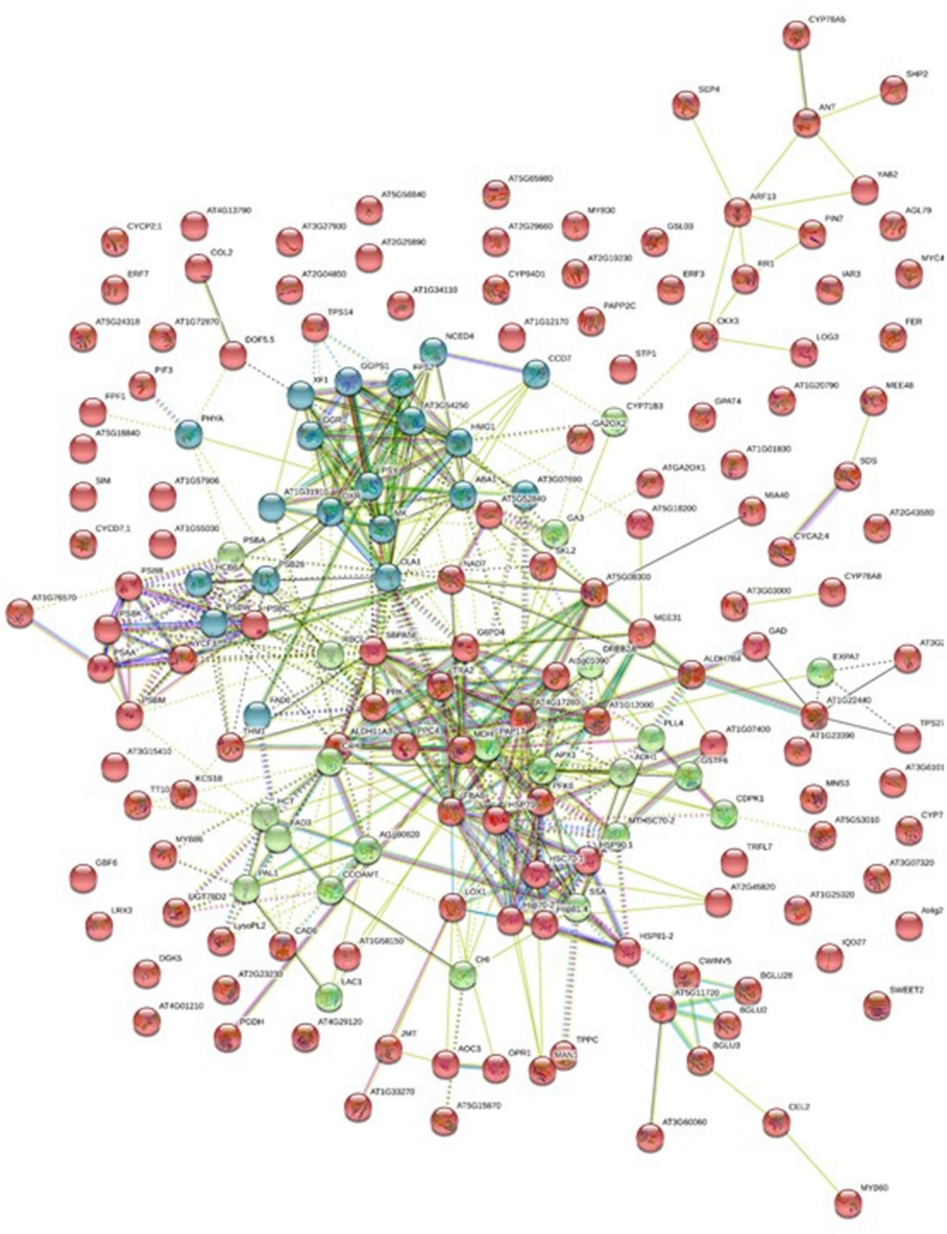
Protein–Protein interaction network of DAPs identified in *N. jatamansi* against known protein-species of Experiment or Text Mining databases. Sky blue lines represent known interactions from curated databases. Purple lines represent experimentally determined known interactions. Green lines represent predicted interactions with neighbourhood genes. Red lines represent gene fusions. Blue lines represent gene co-occurrence. Yellow lines represent text-mining evidence. Black lines represent co-expression. Light blue lines represent protein homology-based interactions. The colored nodes represent query proteins and first shell of interactors, whereas, white nodes represent second shell of interactors. The empty nodes represent proteins of unknown 3D structure, while filled nodes represent proteins with known or predicted 3D structure. Detailed list of the genes predicted in PPi network is given in Supplementary Table 6. Image generated by String v 11.0 (STRING: https://string-db.org).

A total of 318 proteins encoded by DEGs related to plant growth and stress response were identified in transcriptome analysis. These were mapped against STRING database of *Arabidopsis thaliana* and a network of 179 nodes and 472 edges at PPi enrichment *p*-value < 1.0e^−16^ were recorded. The interaction network revealed significant enrichment of GO terms with 177 under biological process, 52 in molecular function terms, 32 in cellular component terms and 29 in KEGG pathways (Fig. 3 and Supplementary Table 6). A strong interaction between the light harvesting genes and the genes of photosynthesis, secondary metabolism (i.e., terpenoid and phenylpropanoid) and genes of stress response i.e., dehydration responsive element binding transcription factor (DREB), heat shock proteins (HSPs) was recorded. Red coloured module represented interactions mainly, in two large clusters followed by 3–4 small clusters of interaction networks. Cluster one showed strong interaction between DAPs of light harvesting complex with DAPs of photosystems. Cluster two revealed multiple strong interactions with the DAPs like 1-deoxy-d-xylulose 5-phosphate reductoisomerase (DXR), mevalonate kinase (MK), phytoene synthase (PSY), omega-6-fatty acid desaturase (FAD6), cinnamate 4-hydroxylase (C4H), geranylgeranyl reductase (GGR), geranylgeranyl pyrophosphate synthase (GGPS1), 9-cis-epoxycarotenoid dioxygenase 4 (NCED4), carotenoid cleavage dioxygenase 7 (CCD7), ABA1 involved in terpenoid, carotenoid and ABA biosyntheses. Blue colored module revealed strong interactions between the 18 DAPs which included sedoheptulose-1,7-biphosphatase (SBPASE), malate dehydrogenase, pyruvate dehydrogenase, phosphoribulokinase, phosphoglycerokinase, phosphofructokinase, fructose biphosphate aldolase, lipoxygenase, aldehyde dehydrogenase (ALDH), glucose 6-phosphate dehydrogenase 4 (G6PD4), and HSPs. Green colored module revealed strong interactions between 22 DAPs of mainly phenylpropanoid biosynthesis pathway and included hydroxycinnamoyl transferase (HCT), phenylalanine ammonia-lyase 1 (PAL1), FAD, C4H, caffeoyl-CoA O-methyltransferase (CCOAMT), chalcone-flavonone isomerase (CHI), laccase, omega-3 desaturase, DREB and ribulose biphosphate carboxylase (RBCL). The RBCL of green module showed strong multiple interactions with ascorbate peroxidase of the same nodule and also with DAPs of photosynthesis of red and phosphoribulokinase of blue module. Lipoxygenase of blue colored module showed interaction with allene oxide cyclase (AOC), jasmonate O-methyltransferase (JMT) and 12-oxophytodienoate reductase (OPR) of red module. The AOC further showed strong interaction with JMT on one hand and OPR on the other.

**Figure 4:**
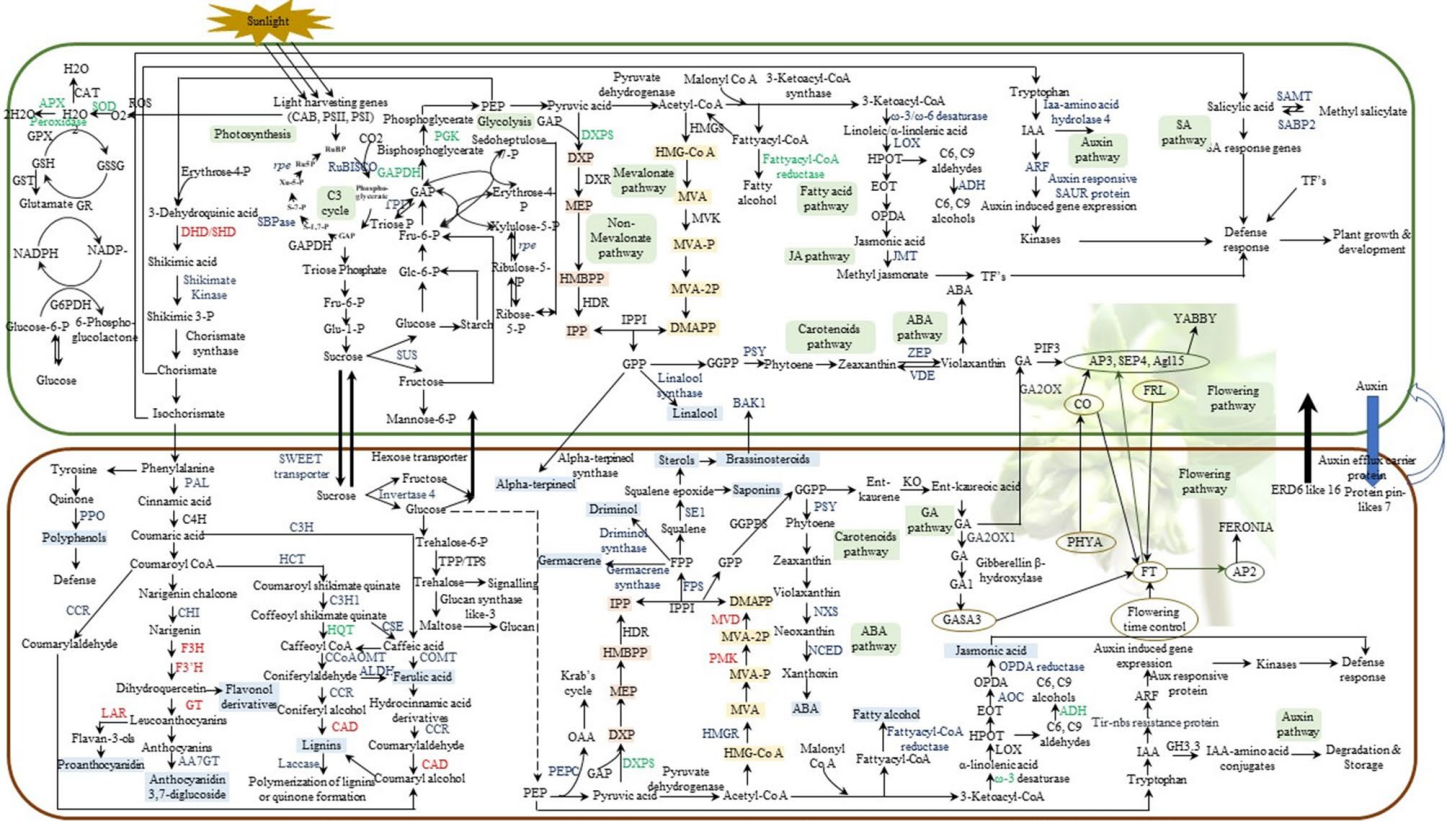
A schematic representation of processes operational in *N. jatamansi* during flowering at the end of growth period. Genes represented in blue colour are upregulated in the same tissue, red colour are upregulated in the alternate tissue. Genes coloured in green are upregulated in both the tissues. Detailed list of names of the genes predicted in the scheme with abbreviations used in the figure is given in Supplementary Table 7.

Based on the differentially expressed genes identified in the leaves and rhizomes of *N. jatamansi* (Supplementary Table 7), a probable genetic machinery underlying various processes (photosynthesis, carbon metabolism, transport of assimilates, flowering, rhizome growth and fortification, stress response and secondary metabolite biosynthesis) occurring in the plant during the end of growth period was proposed (Fig. 4).

### qRT-PCR validation of RNA-sequencing data

**Figure 5:**
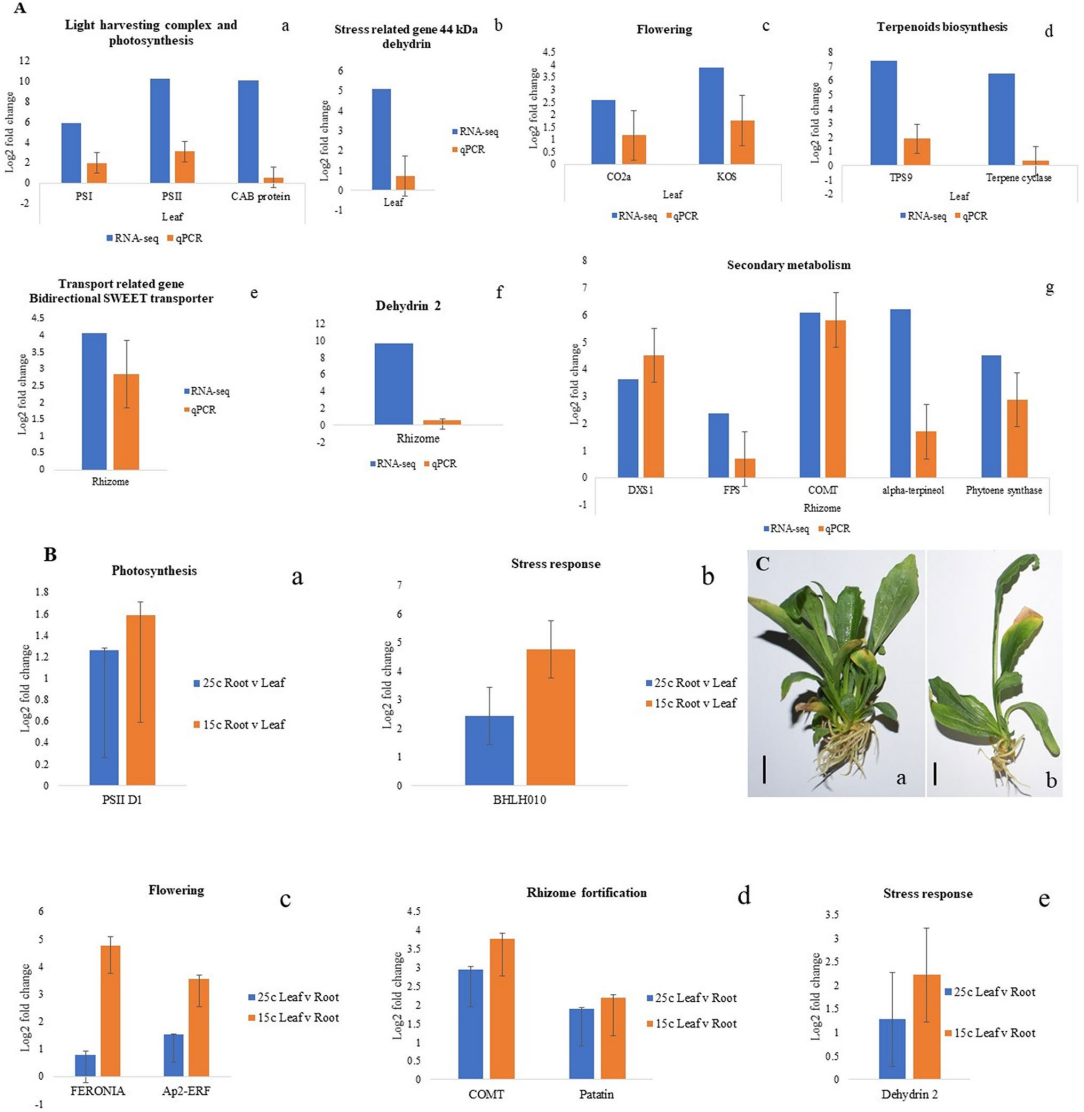
(**A**) qRT-PCR validation of genes involved in growth and adaptation of *N. jatamansi* (a–d) leaves and (e,g) rhizomes. PSI (Photosystem I reaction center subunit XI, chloroplastic), PSII (Photosystem II protein D1), CAB protein (Chlorophyll a-b binding protein, chloroplastic), 44 kDa dehydrin (44 kDa dehydrin-like protein), CO2a (Putative CONSTANS interacting protein 2a-like), KOS (Ent-kaurene oxidase), TPS9 (Terpene synthase), Terpene cyclase (Terpene cyclase/mutase), Bidirectional SWEET transporter, Dehydrin 2, DXS1 (1-deoxy-d-xylulose-5-phosphate synthase 1), FPS (Farnesyl pyrophosphate synthase), COMT (Caffeic acid O-methyl transferase), Alpha-terpineol ((-)-alpha-terpineol synthase), Phytoene synthase; (**B**) qRT-PCR validation of genes involved in growth, reproduction and secondary metabolite synthesis in in vitro grown *N. jatamansi* plants at 15 °C and 14 h/10 h light–dark regime and 25 ºC and 16 h/8 h light–dark regime (a,b) Root v Leaf and (c–e) Leaf v Root; (**C**) In vitro plants of *N. jatamansi* growing at (a) 25 °C and 16 h/8 h light–dark and (b) 15 °C and 14 h/10 h light–dark regimes. Bar = 1 cm.

The reliability of the RNA-seq data was confirmed through qRT-PCR expression analysis of 15 genes (Fig. 5A). The genes of light harvesting such as those encoding PSI, PSII and CAB protein, the water stress related, 44 kDA dehydrin, the flowering related CO2a and KOS, and TPS9 and terpene cyclase of terpenoids biosynthesis were up-regulated in the leaves. The expression pattern of these genes matched perfectly with that of RNA-seq data confirming the simultaneous operation of various processes like light harvesting, flowering and biosynthesis of secondary metabolites during August, the month when the vegetative growth ended. Similarly, perfect matches of the upregulated expression of the bidirectional SWEET transporter, the stress related, dehydrin 2 and the secondary metabolite genes, DXS1, FPS, COMT, α-terpineol and phytoene synthase confirmed the transport of leaf photosynthates to rhizomes and consequent biosynthesis of phenolics, carotenoids and terpenoids in the organ.

### Validation of tissue-specific-expression-of-genes involved in growth reproduction and secondary metabolite synthesis in in vitro grown plants

Environment and tissue specific expression of various genes involved in growth, reproduction and secondary metabolite synthesis was evident from qRT-PCR analysis of in vitro plants growing at two different photoperiods and temperature regimes (representing the peak and end of growth periods). While Photosynthesis II protein D1 and bHLH010 were significantly up-regulated in leaves, COMT, Patatin, Feronia, AP2-ERF and Dehydrin 2 were up-regulated in below-ground parts (Fig. 5B). Interestingly, all of these genes were significantly upregulated at 15 °C and 14 h/10 h light–dark regime as compared to 25 ºC and 16 h/8 h light–dark photoperiod.

### UPLC-PDA analysis of phenolic acids

A total of 6 and 5 phenolics were recorded in the roots of in vitro raised plants at 15 and 25 °C, respectively. Only 3 phenolics i.e., gallic acid, p-coumaric acid and rutin were recorded in the leaves at both 15 and 25 °C (Table 1 and Supplementary Fig. 8).

**Table 1:** UPLC-PDA analysis of phenolics in leaves and roots of in vitro plants of *N. jatamansi.*

Sr no	Tissue	Condition (temperature and photoperiod)	Gallic acid (µg/100 mg FW)	Cinnamic acid (µg/100 mg FW)	P-Coumaric acid (µg/100 mg FW)	Caffeic acid (µg/100 mg FW)	Ferulic acid (µg/100 mg FW)	Kaempferol (µg/100 mg FW)	Rutin (µg/100 mg FW)
1	Roots	15 °C; 14/10 h	5.62 ± 0.04^d^	1.45 ± 0.00^a^	10.58 ± 0.03^a^	4.00 ± 0.05^b^	0.00	1.23 ± 0.00^b^	2.29 ± 0.00^d^
		25 °C; 16/8 h	6.48 ± 0.06^c^	0.00	0.00	8.06 ± 0.04^a^	0.15 ± 0.01	1.94 ± 0.00^a^	9.09 ± 0.02^b^
2	Leaves	15 °C; 14/10 h	16.98 ± 0.03^a^	0.00	7.62 ± 0.01^b^	0.00	0.00	0.00	27.33 ± 0.62^a^
		25 °C; 16/8 h	12.73 ± 0.04^b^	0.00	7.49 ± 0.00^c^	0.00	0.00	0.00	5.17 ± 0.08^c^

Values are mean ± SE from 3 replicates; Different superscript lowercase letters indicate significant difference among treatments (Duncan’s multiple range test *p* ≤ 0.05).

With respect to temperature and plant part, the phenolic contents of roots were higher at 25 °C. Interestingly, the contents of *p*-coumaric and cinnamic acids were very high at 15 °C but they remained undetected at 25 °C. In case of leaves, the contents of gallic acid (16.98 ± 0.03 µg/100 mg FW) and rutin (27.33 ± 0.62 µg/100 mg FW) were significantly higher at 15 ºC, while p-coumaric acid content was at par at both 15 and 25 ºC (7.62 ± 0.01 and 7.49 ± 0.00 µg/100 mg FW, respectively). Irrespective of temperature, caffeic, ferulic and cinnamic acids, and kaempferol remained undetected in the leaves.

## Discussion

As envisaged, the de novo transcriptome analysis of leaves and rhizomes of flowering plants of *N. jatamansi* confirmed the simultaneous operation of various processes like photosynthesis, carbon metabolism, transport of assimilates, flowering, rhizome growth and fortification, stress response and also secondary metabolite biosynthesis during August, the end of growth period (Fig. 4). In the study, DEGs of photosynthesis, generation of precursor metabolites and energy metabolism in leaves; but DEGs of organonitrogen metabolism, cellular carbohydrate metabolism, cellulose and glucan catabolism, cellular polysaccharide metabolism, lignin and peptide biosynthesis, stress response and biosynthesis of metabolites related to phenylpropanoid pathway in rhizomes were significantly enriched in GO analysis (Fig. 1 and Supplementary Fig. 7a–f). Even KEGG analysis revealed the enrichment of carbon and amino acid metabolism, signal transduction and stress response (Supplementary Fig. 4). The dominance of TF families related to growth, flowering, stress response and secondary metabolism also indicated the simultaneous operation of all these processes during the end of growth period. It was quite evident from these results that the assimilates produced in the leaves were being transported to the below-ground parts to support various processes required for the adaptation of *N. jatamansi* to freezing stress during winter. In PPi network also, a strong interaction between the proteins of metabolic pathways like photosynthesis, carbon and fatty acid metabolism and secondary metabolite biosynthesis was recorded.

In RNA-seq data as well as qRT-PCR expression analysis, several genes of light harvesting (chlorophyll a-b binding and also photosystems I and II reaction centre proteins) were highly up-regulated in the leaves (Fig. 5A). It was evident that even during the end of growth period in late August, when the air temperatures and the daylight hours started declining, the leaves harvested maximum light for carbohydrate biosynthesis, transport and allocation of sucrose to plant parts, and finally the conversion of sugars into storage reserves in below-ground parts. In addition to light harvesting, genes involved in carbohydrate synthesis (sedoheptulose-1,7-bisphosphatase, ribulose phosphate 3-epimerase and sucrose synthase) and metabolism (glucose-1-phosphate adenylyl transferase and galactan beta-1,4-galactosyl transferase) were up-regulated in the leaves. The up-regulation of the bidirectional SWEET transporter, the sugar transporter, ERD6-like 16 and glucan synthase like-3, invertase and trehalose 6-phosphate phosphatase in the rhizomes indicated transport of sugars to below-ground parts probably for their accumulation, storage as well as fortification during August, the end of growth period.

Since the plants tested in the present study were in a state of flowering, a number of key unigenes related to all the four flowering pathways (i.e., GA regulated, photoperiodic, autonomous and vernalization) were up-regulated in *N. jatamansi* plant parts. This indicated the participation of both the leaves and rhizomes in the process of flowering. In this regard, Gibberellin regulated protein 3, Gibberellin beta hydroxylase and Gibberellin 2-beta dioxygenases of gibberellin mediated and Phytochrome A of photoperiodic regulation of autonomous pathways were upregulated in the rhizomes. While gibberellin 2-oxidase involved in GA biosynthesis was upregulated in both leaves and rhizomes, putative CONSTANS interacting protein 2a-like (CO) and the zinc finger CONSTANS-LIKE 2 protein of photoperiodic pathway were upregulated in the leaves only. GA related genes interact with floral integrators, while CO activates FT^5^. Each of these separately interact with the floral meristem identity genes namely, AP2, Sepallata and Agamous for flowering^6–8^. Amongst these however, Sepallata 4 (SEP4) and Agamous like-15 (MADS-box protein) were upregulated in the leaves, whereas, only AP2 was upregulated in rhizomes. Moreover, the Flowering Promoting Factor 1, bZIP transcription factor 11 and Feronia in rhizomes but BEL1-like homeodomain (BLH/BELL) and YABBY in leaves were upregulated.

Despite supporting the costly process of flowering, leaves appeared to nurture rhizome growth and fortification. In this regard, the MADS-box transcription factors known to promote storage organ development^9,10^ and the BEL1-like homeodomain (BLH/BELL) known to govern meristem formation, tuberization, rhizome growth, flowering and also ovule morphology^11,12^ were upregulated in leaves. Genes of fatty acid metabolism i.e., omega-6 fatty acid desaturase and jasmonic acid methyl transferase in leaves, 3-ketoacyl-CoA synthase, allene oxidase cyclase and 12-oxophytodienoate reductase, fatty acyl-CoA reductase, glycerol-3-phosphate 2-o-acyltransferase 4 and glycerol-3-phosphate dehydrogenase in rhizomes, and omega-3 fatty acid desaturase and lipoxygenase in both leaves and rhizomes were up-regulated. Fatty acids and lipids are usually stored in perennating below-ground parts of alpine plants for adaptation to cold stress and also for opportunistic growth during the following favourable season after winter^13–15^. Many of these genes, particularly, 12-oxophytodienoate reductase participate in signalling via jasmonic acid for acclimation of plants under cold stress^16^. Signalling mediated adaptation to alpine habitat was also evident from the up-regulation of Tir-nbs resistance protein in *N. jatamansi* rhizome. Stress responsive genes such as HSP20, 70, 90, 24, expansin, patatin and Dof 2.1 were also up-regulated in rhizomes. The heat shock proteins, expansin, patatin and also the transcription factors namely, Dof 2.1, ERF, C2H2 and DREB are known to impart cold/freezing stress tolerance in plants^17–19^.

Partitioning of a part of the leaf photosynthates (erythrose 4 phosphate) towards the biosynthesis of shikimic acid and phenyl propanoids was indicated by the upregulated unigenes of these pathways in both leaves and rhizomes. The up-regulation of the unigenes of glycolysis and those of MEP and MVA pathways indicated a distinct diversion of the end products of glycolysis towards the biosynthesis of fatty acids, terpenoids, phyto-hormones (abscisic acid and gibberellins) and the signalling molecule, jasmonic acid (Supplementary Table 7).

Interestingly, genes of only monoterpene biosynthesis in leaves (> 10 log2 fold change), but di and sesquiterpene in rhizomes were highly up-regulated (> 7 log2 fold change). Genes of squalene epoxide (SE1), PSY, neoxanthin epoxidase (NXS), NCED1 and also those of polyphenols, flavan-3-ols and anthocyanins biosynthesis in rhizomes, but PSY, zeaxanthin epoxidase (ZEP) and violaxanthin de epoxidase (VDE) in leaves were upregulated (Fig. 4; Supplementary Table 7). SE1 is known for its role in the biosynthesis of saponins, sterols and brassinosteroids^20,21^, whereas, those of PSY leads to carotenoid biosynthesis^22^. Moreover, this, along with ZEP, VDE, NXS and NCED lead to the biosynthesis of ABA^23,24^. Based on these observations, a scheme on simultaneous utilization of leaf photosynthates for various processes was developed. The qRT-PCR analysis of in vitro plants growing at two different temperatures and photoperiods (representative of peak and end of growth periods) validated the RNA-seq data. It also revealed invariably higher gene expression during declining air temperature and photoperiod (Fig. 5C). Moreover, environment- and tissue- specific expression of genes related to growth, reproduction, stress response and secondary metabolite biosynthesis were upregulated during the end of growth period (Fig. 5B).

UPLC-PDA analysis of the phenolic acids also revealed their tissue and temperature specific distribution (Tables 1 and 2). Invariably, higher number of phenolics were recorded in the roots at 15 and 25 ºC as compared to leaves, thereby, indicating a higher inclination of secondary metabolites towards underground parts as compared to aerial parts. In case of gene expression analysis (RNA-seq data) also, higher number of genes related to phenylpropanoid pathway were upregulated in below-ground parts. The phenolics recorded in the study are known to impart protection against UV radiation and pathogens in plants^25^. Their upregulation in *N. jatamansi* is probably an important requisite for their growth and survival in the plant’s native alpine habitats. Thus, *p-*coumaric and cinnamic acids known for their role in lignin biosynthesis were significantly high in the roots at 15 °C.

**Table 2:** Beneficial effects of different phenolics detected in in vitro plants of *N. jatamansi* in UPLC-PDA analysis.

S. no.	Compound name	Use in plant	Health benefits/accounts for
1	Gallic acid	Protection against UV and pathogens^39^ Cellular communication and signalling^25^	Anti-inflammatory, antimutagenic, antifungal, antiviral, anticancer and antioxidant activities^40^
2	Cinnamic acid	UV protectant and defence against pathogens^39^, lignification of cell wall^41^ Cellular communication and signalling^25^	Anticancer, antituberculosis, antimalarial, antifungal, antimicrobial, antiatherogenic and antioxidant activities^42^
3	p-Coumaric acid	UV protectant and defence against pathogens^39^ Cellular communication and signalling^25^	Anti-melanogenic, anti-inflammatory, anti-cancerous activities^43^
4	Caffeic acid	UV protectant and defence against pathogens (insects, fungi and bacteria)^39^ Used for lignin synthesis leading to cell wall thickening Cellular communication and signalling^25^	Antibacterial, antiviral, antioxidant, anti-inflammatory, anti-atherosclerotic, immunostimulatory, antidiabetic, cardioprotective, antiproliferative, hepatoprotective, anticancer and anti-hepatocellular carcinoma activities^44^
5	Ferulic acid	Component of primary cell wall, Cell wall rigidity Cellular communication and signalling^25^ UV protectant and defence against pathogens^39^	Used in skin care products Anti-inflammatory, antioxidant, antimicrobial, anticancer, and antidiabetic, anti-ageing activities^45^
6	Kaempferol	UV protectant and defence against pathogens^46^ Cellular communication and signalling	Anti-inflammatory, anti-tumour, anti-oxidant, cardiovascular, antidiabetic, hepatoprotective and neuroprotective effects^47^
7	Rutin	UV protectant and defence against pathogens^48^	Anti-inflammatory, antioxidant, cytoprotective, vasoprotective, anticarcinogenic, neuroprotective, antibacterial, antiprotozoal, antitumor, antiallergic, antiviral, vasoactive, cardioprotective, hypolipidaemic, antiplatelet, antispasmodic, and antihypertensive activities^49,50^

*Nardostachys jatamansi* is known to have a wide array of medicinal properties that had remained unaccounted for till date^3^. Up to now, terpenoids mainly, sesquiterpenes were considered to be the major group of secondary metabolites responsible for the various medicinal properties of *N. jatamansi*. However, the phenolic compounds which were identified in the plant in the present study are known to have a number of health endowing properties in humans (Table 2). Thus, it is apparent from these findings that many of the therapeutic properties of *N. jatamansi* are probably attributable to the phenolic compounds recorded in this study. Probably, these phenolic compounds account for the various pharmacologically important properties reported in *N. jatamansi.*

## Conclusion

The study is the first report on the de novo transcriptome analysis of *N. jatamansi*. The assumption that even during the costly process of flowering at the end of growth period, leaf photosynthates are utilized for the fortification of rhizomes as adaptive preparation for winter was confirmed. The study also revealed several tissue-specific-secondary-metabolites that have not been reported till date in *N. jatamansi*. The biosynthesis pathway genes of important secondary metabolites identified in the study, showed invariably higher expression at lower temperature (15 °C) and lesser light hours. UPLC-PDA analysis also revealed tissue and temperature specific differential distribution pattern of phenolics in leaves and roots at different temperatures, with a clear bias towards the underground parts. In addition, the phenolics recorded in the plant accounted for a range of medicinal properties of *N. jatamansi*. These findings provided cues for higher secondary metabolite synthesis in alternative in vitro systems. Since, it can eliminate the need for uprooting *N. jatamansi* from wild, the study is a step towards effective conservation of this high value, critically endangered alpine herb.

## Materials and methods

### Plant material

*Nardostachys jatamansi* plants growing at Tandi, Lahaul & Spiti, Himachal Pradesh, India (latitude 32° 33′ 53″ N; longitude 76° 58′ 05″ E and altitude 3040 m amsl) were selected for the study. Leaf as well as rhizome samples of the plant were collected during August (peak flowering) and frozen immediately in liquid nitrogen. These were then stored at − 80 °C for further use.

### RNA library preparation

The iRIS method of Ghawana et al.^26^ was used to extract total RNA from the above-mentioned samples (three biological replicates of each of leaf and rhizome sample). While the quality and quantity of the total RNA were analyzed using Nanodrop spectrophotometer, a bioanalyzer (Agilent 2100 technologies, USA) was used for further evaluation.

### Transcriptome sequencing

Equal amounts of RNA from leaf and rhizomes (biological samples) in triplicate were pooled together. Finally, 5.0 µg RNA from the pool was processed for library preparation using TruSeq RNA sample Prep Kit v2 (Illumina Incorporation, USA) according to manufacturer’s instructions manual. The libraries were quantified using Qubit dsDNA BR assay kit for Qubit 2.0 Fluorometer (Life technologies, USA). After checking the insert size of the libraries using a bioanalyzer DNA 1000 chip, 10 pM of each library was loaded onto the flow cell using TruSeq PE Cluster Kit v5 on cluster station (Illumina Inc., USA). The flow cell containing the clonally amplified clusters was then loaded onto the Genome Analyzer IIx (Illumina) for paired-end (PE) (2 × 72) sequencing. The GERALD base-calling (a CASAVA package total provided by Illumina) was used to transform the raw sequencing data into ‘single end’ (SE) 72 bp reads. The resulting sequence reads were stored in FASTQ format and the raw sequences submitted to NCBI SRA database (Accession no. PRJNA608834). After checking the quality of the sequence reads by Fast QC^27^, the quality reads were filtered using the Cutadapt-1.8.3 tool^28^. While the adapter sequences, reads of low quality and also very short lengths were removed, the low quality bases were trimmed at 3′-end.

### De novo data assembly

Multiple assembly tools were checked for de novo assembly of transcriptome data but the Trinity based assembly with K-mer of 25 was selected on the basis of N50 value, number of contigs and read usage (> 200 bp)^29^. The assembled transcripts were then clustered on the basis of 95% similarity between sequences. The reads obtained from each sample were merged using CD-HIT 4.5.4^30^. Unigenes were generated on the basis of best balance between the number of contigs produced, average coverage, N50 value of total assembly and average sequence length. Redundant sets of sequences were removed by filtering, and similar sequence stretches merged using CD-HIT-EST based sequential and hierarchical clustering. CD-HIT-EST with 95% similarity cut-off was also used for the reduction of redundancy between similar types of contigs.

### Functional annotation of the assembled transcriptome

Quality transcripts obtained as above were functionally annotated using multiple databases like Uniprot^31^, Gene Ontology (GO), KAAS^32^ and MISA^33^. BLAST-2.5.0^30^ was also used for annotations of transcripts. Homology search was performed against *Viridiplanteae* datasets from Uniprot database. E-value distribution and species similarities were also performed. Considering the dicot model organisms as ‘reference’ for pathway identification, the KAAS server was used for KEGG orthology assignment of transcripts.

### SSR prediction

From among the assembled sequences, the status of SSRs in the transcript sequences were predicted using MISA Perl^33^ because of the software’s ability to predict ‘perfect SSRs’ as well as ‘compound SSRs with spacer sequences, un-interruptedly’. The Fasta files containing the assembled sequences were then used as input files. The minimum number of repeats for microsatellites (unit size/minimum number of repeats) were specified as 2/6, 3/5, 4/5, 5/5 and 6/5; and a variable of 100 bp was set to specify the maximum length of the spacer sequences.

### Differential expression analysis

Unigenes representing the assembled transcripts of each biological sample was generated by CD-HIT clustering, and sequences with < 200 nucleotides were omitted by filtering. Bowtie2 was used to separately map the reads from each plant part onto unigenes^34^. The expression of unigenes were also quantified. The sequencing bias among the biological samples was avoided by edgeR in pair-wise comparisons^35^. The differentially expressed genes between rhizomes and leaves were finally analysed and genes having log_2_ fold change value of more than ‘2’ were considered as upregulated. Genes with log_2_ fold change less than ‘2’ were considered as down-regulated.

### GO enrichment analysis

GO enrichment analysis of DEGs from leaves and rhizomes was performed using AgriGOv2.0^36^ (https://systemsbiology.cau.edu.cn/agriGOv2/c_SEA.php). The GO enriched categories were identified using the Hygrogeometric statistical test at 0.05 and the Bonferroni multi-test adjustment method. The transcription factors and their families were identified using Plant TFDB v 5.0^37^ (https://planttfdb.cbi.pku.edu.cn/).

### Protein–protein interaction (PPI) network

The interactions between DEGs of leaves and rhizomes related to growth, development, adaptation and secondary metabolite biosynthesis were mapped against STRING database of *Arabidopsis thaliana*. A protein–protein interaction (PPI) network of the resulting genes/proteins were constructed using STRING v11.0 (the Search Tool for the Retrieval of Interacting Genes/Proteins)^38^.

### Quantitative real time PCR

Based on the above findings, genes related to growth and development, secondary metabolite biosynthesis and adaptation were selected for the validation of RNA-seq data. Total RNA from triplicate samples of each of leaf and rhizome were isolated as described above. The Verso cDNA synthesis kit (Thermo Scientific, USA) was used to synthesise the first stand cDNAs. Primers for each of the selected genes were designed using primer express 3.0 software (Supplementary Table 8). Finally, qRT-PCR of each sample was performed in triplicate using the ABI StepOne Real time PCR machine of Applied Biosystems, Thermofisher Scientific, USA. A 10 μl reaction mixture comprising of 5.0 μl of 2X SYBR Green Master Mix, 0.2 μM gene specific forward and reverse primers and template cDNA at 1:10 dilution was used. The PCR amplification cycle comprised of an initiation step at 95 °C for 20 s followed by 40 cycles of amplification at 95 °C for 15 s, 60 °C for 60 s; melting curve of 95 °C for 15 s, 60 °C for 60 s and finally, 95 °C for 15 s. The relative quantification method (2^−ΔΔCT^) was used to evaluate the quantitative variations between replicates. Actin was used as internal control.

### Validation of genes related to growth reproduction and secondary metabolite synthesis in in vitro growing shoot cultures

In vitro plants of *N. jatamansi* (3.0 cm) were grown for 30 days at two different temperatures and photoperiods, i.e., (i) 16 h light (70 μmol m^−2^ s^−1^ from Philips white fluorescent tubes)/8 h dark at 25 ºC (representing the temperature during optimal vegetative growth period); and (ii) 14 h light (70 μmol m^−2^ s^−1^ from Philips white fluorescent tubes)/10 h dark at 15 ºC (representing the temperature during flowering and end of vegetative growth period). After 30 days, total RNA was isolated from 100 mg tissue samples of each of roots and leaves in triplicate. These were then used for cDNA synthesis as described above and validated by qRT-PCR. Primer pairs of genes related to photosynthesis, rhizome formation, flowering and secondary metabolite biosynthesis were used. The details of primer sequence for each gene is given in Supplementary Table 8.

### UPLC-PDA analysis of phenolic acids

#### Sample preparation

Fresh tissues (200 mg) of leaves and roots of in vitro plants growing at two different temperatures and photoperiods, i.e., (i) 16 h light/ 8 h dark at 25 ºC; and (ii) 14 h light/10 h dark at 15 ºC were homogenized in liquid nitrogen to form a fine powder. The samples were extracted with 2.0 ml of 70% methanol by centrifugation for 10 min at 7000 rpm. Extracts were transferred to a fresh centrifuge tube and additional 1.5 ml of 70% methanol was used to recover the left-over sample, twice. Extracts were pooled together and the final volume was made up to 5.0 ml. These were then centrifuged at 7000 rpm for 15 min at 4 ºC. The samples were either stored at 4 ºC or used for UPLC-PDA analysis after filtering through 0.22-µm filter (Millipore, USA).

#### Standard preparation

Standards of phenolic acids namely, gallic acid, caffeic acid, p-coumaric acid, rutin, ferulic acid, quercetin, cinnamic acid and kaempferol were prepared at 1:1 mg/ml (w/v) concentration in HPLC grade methanol. The standards were purchased from Sigma Aldrich, India.

#### UPLC-PDA analysis

Quantification of selected phenolic acids and flavonoids was performed using Waters HSS-T3 C18 column (2.1 mm × 100 mm, 1.8 µm), operated by Waters Acquity UPLC, H-class system (Waters, Milford, MA, USA). The mobile phase consisted of A (water containing 0.1% formic acid) and B (acetonitrile (ACN) containing 0.1% formic acid) in gradient elution. The gradient comprised of 10% B at 0–1 min; and 1–8 min of linear gradient from 10% B to 45% B; 95% B at 8–10 min followed by 10% B at 10–13 min. Detection wavelength was set at 270 nm and the elution was performed using an injection volume of 2.0 µl at a solvent flow rate of 0.25 ml/min. Data was calculated by plotting the calibration curve of selected standards and using regression equation. Data was statistically analysed using SPSS v 14.0 software (SPSS Inc., Chicago, Illinois, USA). The statistical significance between the mean values was assessed by Duncan’s Multiple Range Test (DMRT) at a probability/significance level of P ≤ 0.05.

## Supplementary information

Supplementary Information.

Supplementary Table S1.

Supplementary Table S2.

Supplementary Table S3.

Supplementary Table S4.

Supplementary Table S5.

Supplementary Table S6.

Supplementary Table S7.

Supplementary Table S8.

## Data Availability

All data generated or analysed during this study are included in this published article and its supplementary information files. All the sequencing data have been deposited in the NCBI SRA database having accession no. PRJNA608834.

## References

[1] Chauhan RS, Nautiyal MC (2005). Commercial viability of cultivation of an endangered medicinal herb *Nardostachys jatamansi* DC. at three different agro-climatic zones. Curr. Sci..

[2] Chauhan RS, Kaul MK, Kumar A, Nautiyal MC (2008). Pollination behaviour of *Nardostachys jatamansi* DC., an endangered medicinal and aromatic herb. Sci. Hortic..

[3] Dhiman N, Bhattacharya A (2020). *Nardostachys jatamansi*—The challenges and opportunities of harnessing the untapped pharmaceutical resources. J. Ethnopharmacol..

[4] https://www.nmpb.nic.in/medicinal_list

[5] Yoo SK (2005). CONSTANS activates SUPPRESSOR OF OVEREXPRESSION OF CONSTANS 1 through FLOWERING LOCUS T to promote flowering in *Arabidopsis*. Plant Physiol..

[6] Kania T, Russenberger D, Peng S, Apel K, Melzer S (1997). FPFI promotes flowering in *Arabidopsis*. Plant Cell.

[7] Kurokura T, Mimida N, Battey NH, Hytönen T (2013). The regulation of seasonal flowering in the Rosaceae. J. Exp. Bot..

[8] Yan Q (2019). Comprehensive analysis of bZIP transcription factors uncovers their roles during dimorphic floret differentiation and stress response in *Cleistogenes songorica*. BMC Genomics.

[9] Gao H (2018). Genome-wide survey of potato MADS-box genes reveals that StMADS1 and StMADS13 are putative downstream targets of tuberigen StSP6A. BMC Genomics.

[10] Castelán-Muñoz N (2019). MADS-Box genes are key components of genetic regulatory networks involved in abiotic stress and plastic developmental responses in plants. Front. Plant Sci..

[11] Hannapel DJ (2004). Molecular controls of tuberization. Am. J. Potato Res..

[12] Sharma P (2014). The BEL1-like family of transcription factors in potato. J. Exp. Bot..

[13] Upchurch RG (2008). Fatty acid unsaturation, mobilization, and regulation in the response of plants to stress. Biotechnol. Lett..

[14] Badea C, Basu SK (2009). The effect of low temperature on metabolism of membrane lipids in plants and associated gene expression. Plant Omics.

[15] Barrero-Sicilia C, Silvestre S, Haslam RP, Michaelson LV (2017). Lipid remodelling: Unravelling the response to cold stress in *Arabidopsis* and its extremophile relative *Eutrema salsugineum*. Plant Sci..

[16] Wasternack C, Strnad M (2016). Jasmonate signaling in plant stress responses and development - active and inactive compounds. New Biotechnol..

[17] Liu Q (2000). Regulatory role of DREB transcription factors in plant drought, salt and cold tolerance. Chin. Sci. Bull..

[18] Sun X, Wang Y, Sui N (2018). Transcriptional regulation of bHLH during plant response to stress. Biochem. Biophys. Res. Commun..

[19] Feng X (2019). TaEXPB7-B, a β-expansin gene involved in low-temperature stress and abscisic acid responses, promotes growth and cold resistance in *Arabidopsis thaliana*. J. Plant Physiol..

[20] Zhao CL, Cui ZM, Chen YP, Liang Q (2010). Key enzymes of triterpenoid saponin biosynthesis and the induction of their activities and gene expressions in plants. Nat. Prod. Commun..

[21] Vriet C, Russinova E, Reuzeau C (2013). From squalene to brassinolide: The steroid metabolic and signalling pathways across the plant kingdom. Mol. Plant.

[22] Rodríguez-Villalón A, Gas E, Rodríguez-Concepción M (2009). Phytoene synthase activity controls the biosynthesis of carotenoids and the supply of their metabolic precursors in dark-grown *Arabidopsis* seedlings. Plant J..

[23] Xiong L, Zhu JK (2003). Regulation of abscisic acid biosynthesis. Plant Physiol..

[24] Finkelstein R (2013). Abscisic acid synthesis and response. The Arabidosis Book.

[25] Bhattacharya A, Sood P, Citovsky V (2010). The roles of plant phenolics in defence and communication during *Agrobacterium* and *Rhizobium* infection. Mol. Plant Pathol..

[26] Ghawana S (2011). An RNA isolation system for plant tissues rich in secondary metabolites. BMC Res. Notes.

[27] FastQC: https://www.bioinformatics.babraham.ac.uk/projects/fastqc/

[28] Marcel, M. Cutadapt removes adapter sequences from high-throughput sequencing reads. (2014).

[29] Grabherr MG (2011). Trinity: Reconstructing a full-length transcriptome without a genome from RNA-Seq data. Nat. Biotechnol..

[30] Altschul S (1990). Basic local alignment search tool. J. Mol. Biol..

[31] Uniprot: https://www.uniprot.org/

[32] Moriya Y (2007). KAAS: An automatic genome annotation and pathway reconstruction server. Nucleic Acids Res..

[33] MISA: https://pgrc.ipk-gatersleben.de/misa/

[34] Langmead B, Salzberg SL (2012). Fast gapped-read alignment with Bowtie 2. Nat. Methods..

[35] Robinson MD, McCarthy DJ, Smyth GK (2010). edgeR: A Bioconductor package for differential expression analysis of digital gene expression data. Bioinformatics.

[36] Tian T (2017). agriGO v2.0: A GO analysis toolkit for the agricultural community. Nucleic Acids Res..

[37] Tian F (2020). PlantRegMap: Charting functional regulatory maps in plants. Nucleic Acids Res..

[38] Szklarczyk D (2018). STRING v11: Protein–protein association networks with increased coverage, supporting functional discovery in genome-wide experimental datasets. Nucleic Acids Res..

[39] Lattanzio, V., Lattanzino, V.M.T. & Cardinali, A. Role of phenolics in the resistance mechanisms of plants against fungal pathogens and insects. In *Phytochemistry: advances in research* (ed. Fillippo Imperato) 23–67 (2006).

[40] Borde VU, Pangrikar PP, Tekale SU (2011). Gallic acid in ayurvedic herbs and formulations. Recent Res. Sci. Technol..

[41] Salvador VH (2013). Cinnamic acid increases lignin production and inhibits soybean root growth. PLoS ONE.

[42] Guzman JD (2014). Natural cinnamic acids, synthetic derivatives and hybrids with antimicrobial activity. Molecules.

[43] Boo YC (2019). p-Coumaric acid as an active ingredient in cosmetics: A review focusing on its antimelanogenic effects. Antioxidants.

[44] Espíndola KMM (2019). Chemical and pharmacological aspects of caffeic acid and its activity in hepatocarcinoma. Front. Oncol..

[45] Kumar N, Pruthi V (2014). Potential applications of ferulic acid from natural sources. Biotechnol. Rep..

[46] Samanta A, Das G, Das S (2011). Roles of flavonoids in plants. Int. J. Pharm. Sci. Tech..

[47] Wang J (2018). Antitumor, antioxidant and anti-inflammatory activities of kaempferol and its corresponding glycosides and the enzymatic preparation of kaempferol. PLoS ONE.

[48] Kreft I, Fabjan N, Germ M (2003). Rutin in buckwheat—Protection of plants and its importance for the production of functional food. Fagopyrum.

[49] Sharma S (2013). Rutin: Therapeutic potential and recent advances in drug delivery. Expert Opin. Investig. Drugs.

[50] Huang X (2016). Efficient rutin and quercetin biosynthesis through flavonoids-related gene expression in *Fagopyrum tataricum* Gaertn. Hairy root cultures with UV-B irradiation. Front. Plant Sci..

