# Supplementary material for: De novo transcriptome analysis of the critically endangered alpine Himalayan herb Nardostachys jatamansi reveals the biosynthesis pathway genes of tissue-specific secondary metabolites
Source: Sci Rep. 2020 Oct 14;10:17186. doi: 10.1038/s41598-020-74049-1 (PMC7560736; doi:10.1038/s41598-020-74049-1)
Supplement: Supplementary file 1 — Supplementary Information. [file 41598_2020_74049_MOESM1_ESM.docx]

**Supplementary Information:**

**Original Research Article:**

**Title of the paper**

***De novo* transcriptome analysis of the critically endangered alpine Himalayan herb *Nardostachys jatamansi* reveals the biosynthesis pathway genes of tissue-specific secondary metabolites**

Nisha Dhiman^1,2^, Anil Kumar^3^, Dinesh Kumar^3^, Amita Bhattacharya^1,2*^

^1^Division of Biotechnology, CSIR-Institute of Himalayan Bioresource Technology, Palampur-176061, H.P., India

^2^Academy of Scientific and Innovative Research, CSIR-Institute of Himalayan Bioresource Technology, Palampur-176061, H.P., India

^3^Natural Product Chemistry and Process Development Division, CSIR-Institute of Himalayan Bioresource Technology, Palampur-176061, H.P., India

*Corresponding Author

*E-mail address*: [amitabhatta@ihbt.res.in](mailto:amitabhatta@ihbt.res.in); [amitabhatta@yahoo.uk.co.in](mailto:amitabhatta@yahoo.uk.co.in) and ORCID ID: [orcid.org/0000-0003-1936-2995](https://orcid.org/0000-0003-1936-2995#_blank)

**Supplementary Figures:**

**Supplementary Fig. 1:** Clustering of transcripts of leaves and rhizomes of *N. jatamansi* as per their lengths in base pair


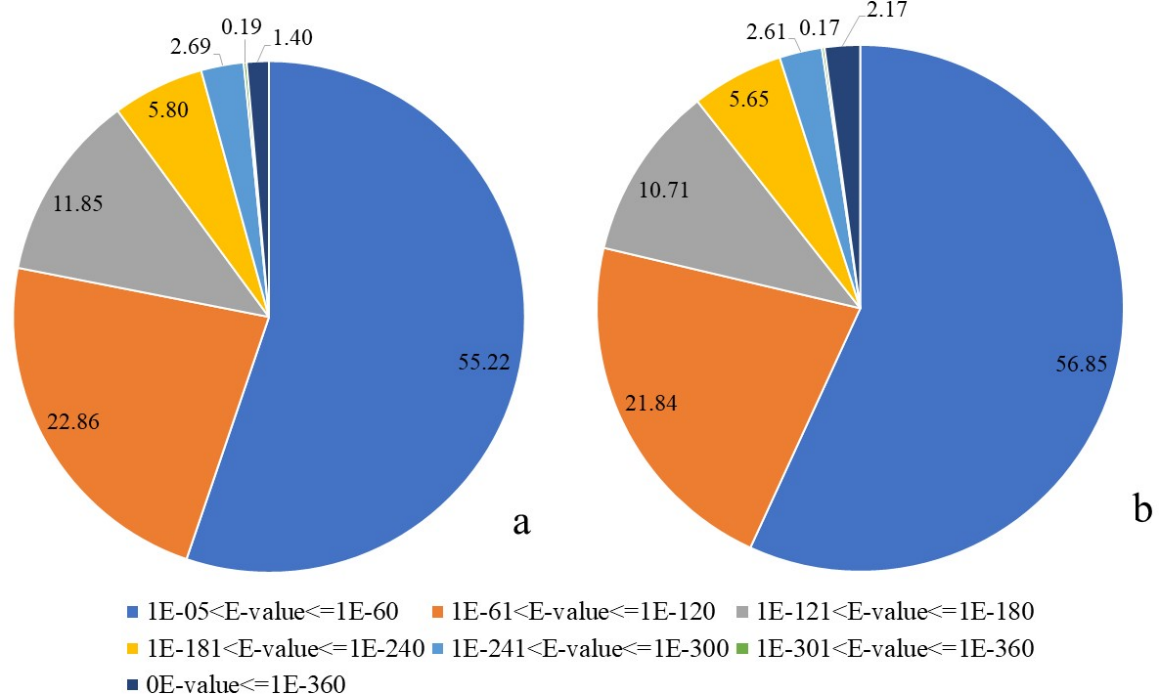


**Supplementary Fig. 2:** Percent distribution of annotated transcripts of a) leaves and b) rhizomes of *N. jatamansi* based on E-value

**Supplementary Fig. 3:** Distribution of transcripts of leaves and rhizomes of *N. jatamansi* based on species similarity


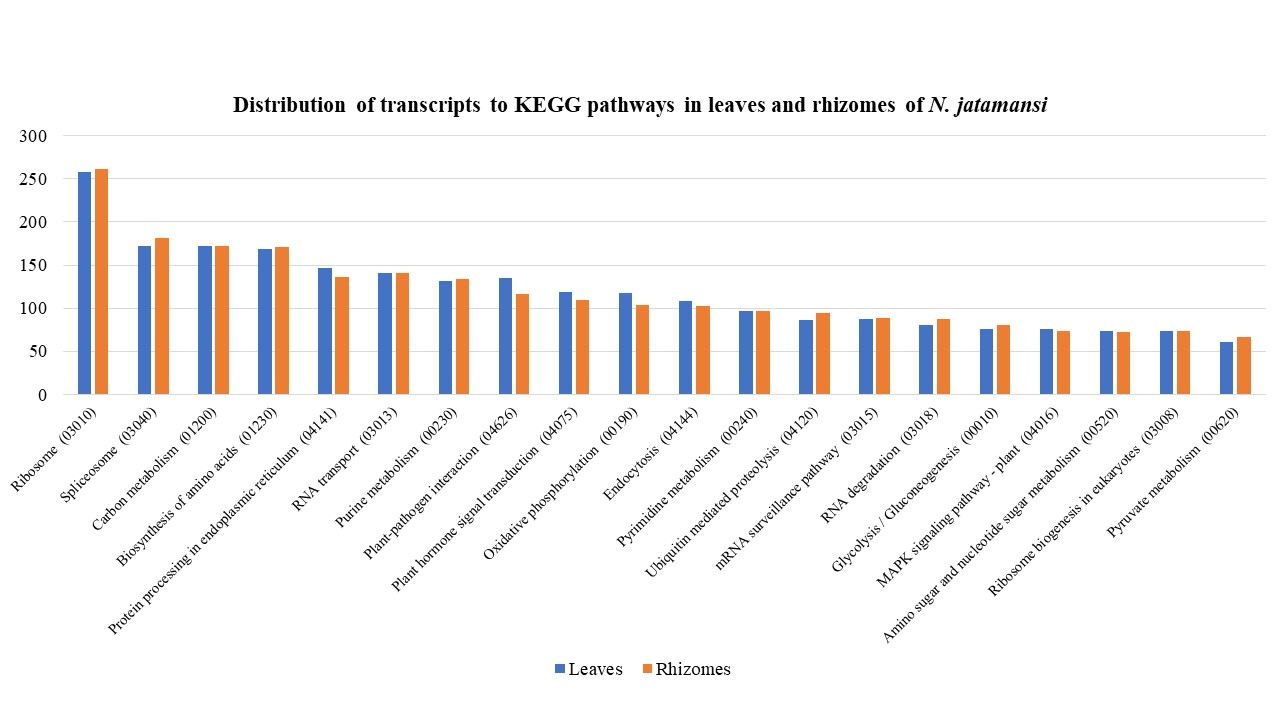


**Supplementary Fig. 4:** Top 20 KEGG pathways in *N. jatamansi* leaves and rhizomes


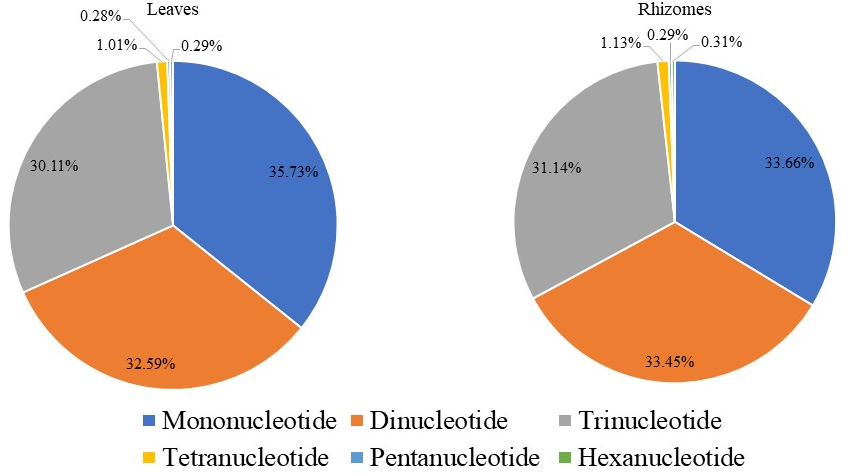


**Supplementary Fig. 5:** Different classes of SSRs identified in leaves and rhizomes of *N. jatamansi*

**Supplementary Fig. 6:** Details of upregulated transcripts from leaves and rhizomes of *N. jatamansi*. LvR_Up represents upregulation in rhizomes as compared to leaves and RvL_Up represents upregulation in leaves as compared to rhizomes


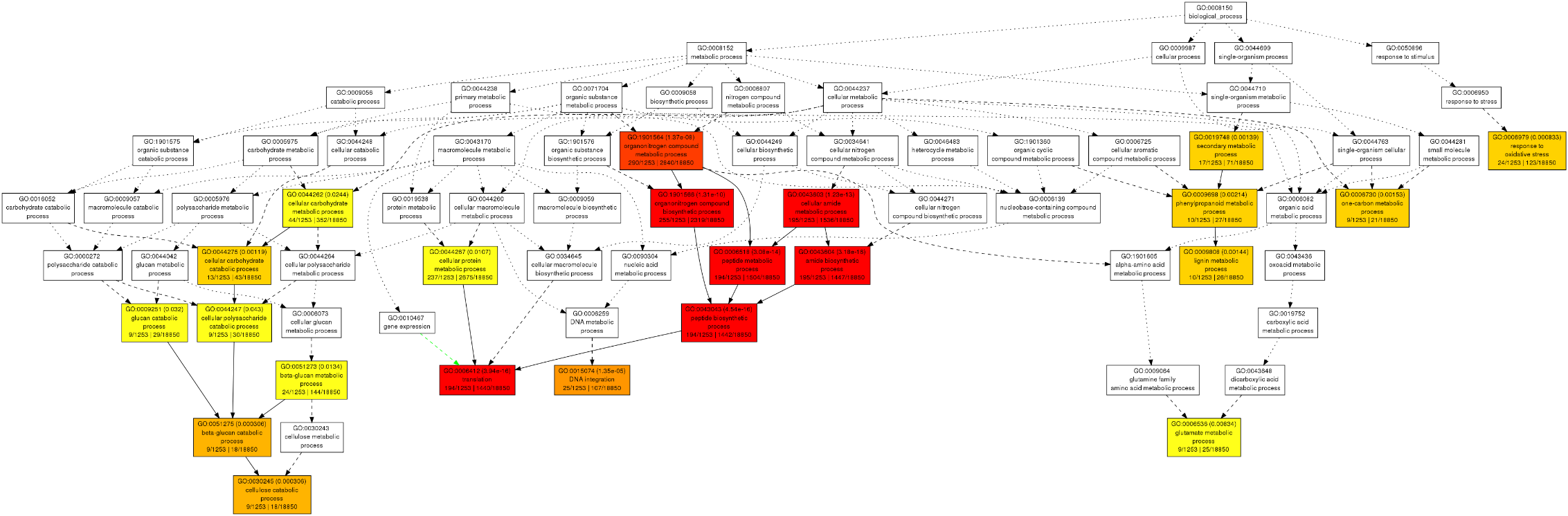


**Supplementary Fig. 7(a): Upregulated GO enriched categories of rhizomes as compared to leaves under biological processes**


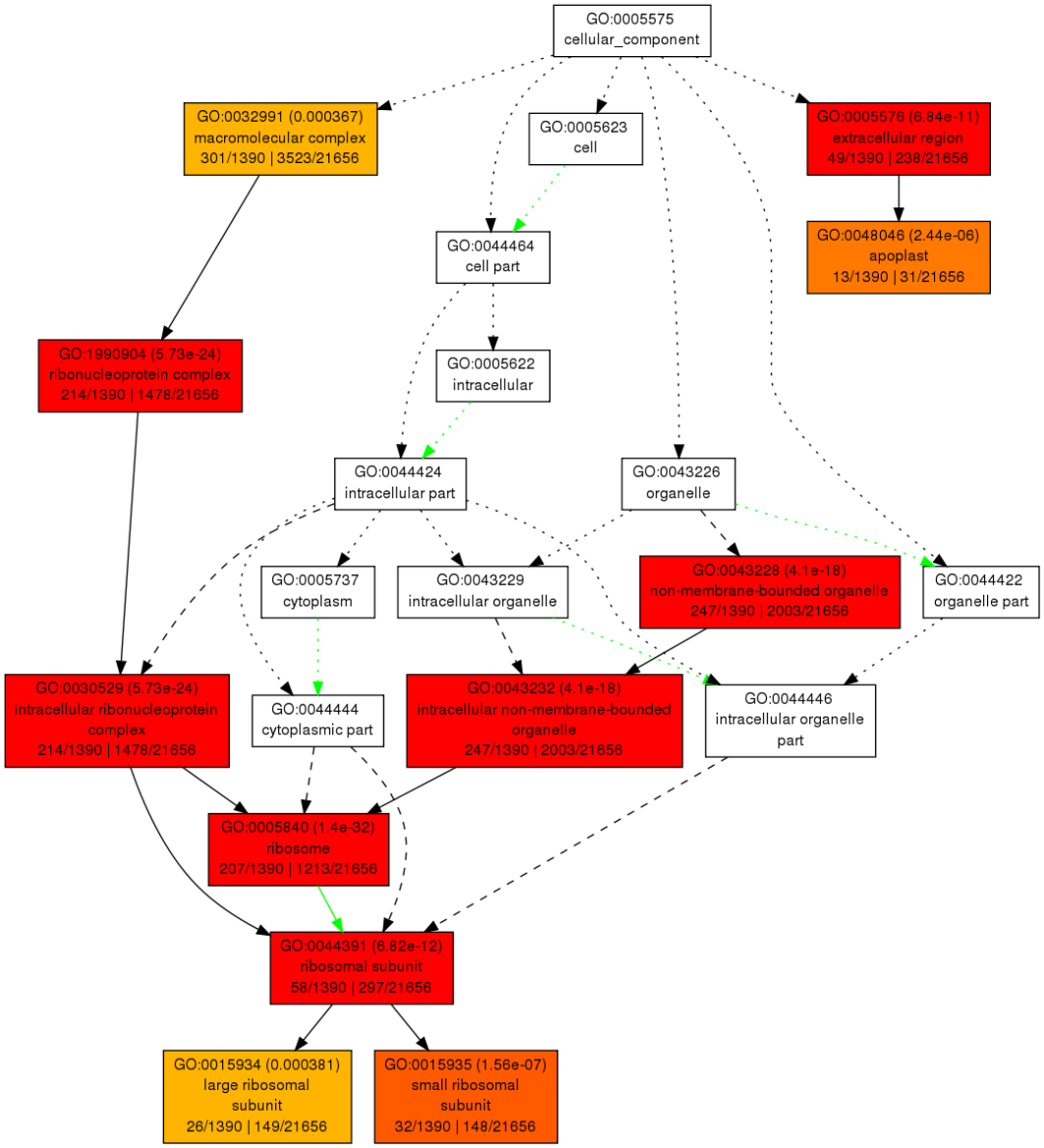


**Supplementary Fig. 7(b): Upregulated GO enriched categories of rhizomes as compared to leaves under cellular components**


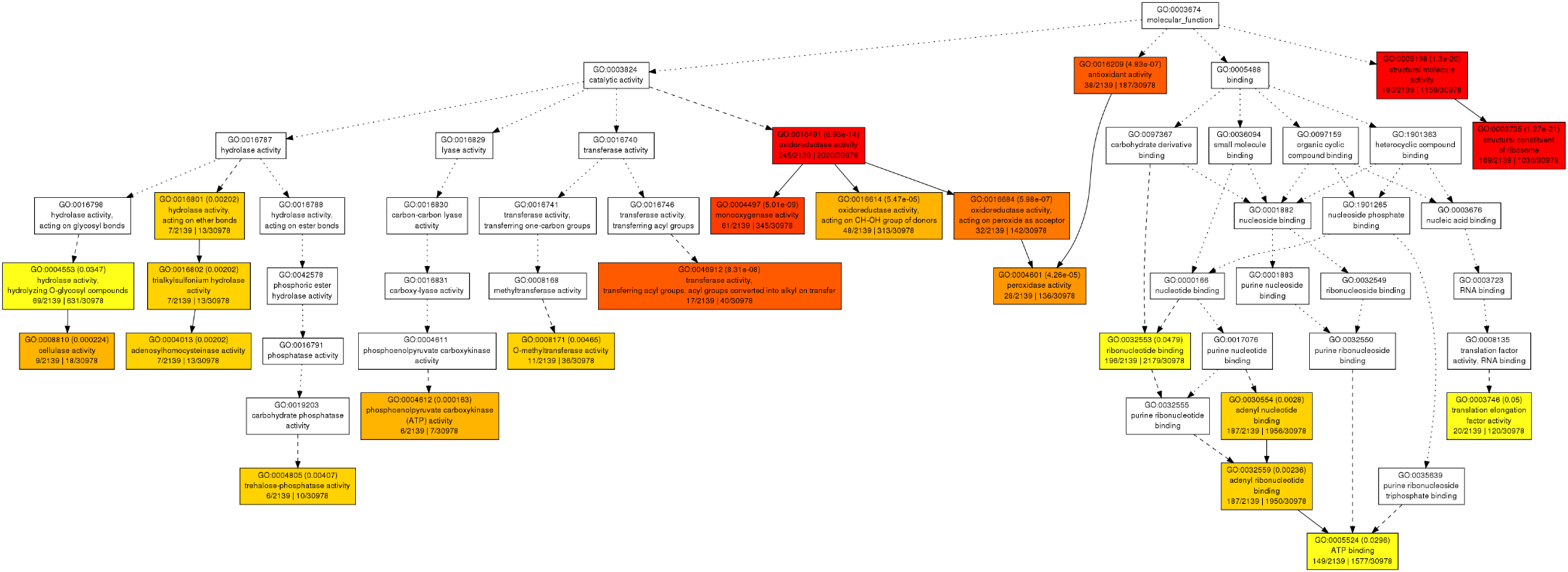


**Supplementary Fig. 7(c): Upregulated GO enriched categories of rhizomes as compared to leaves under molecular function**


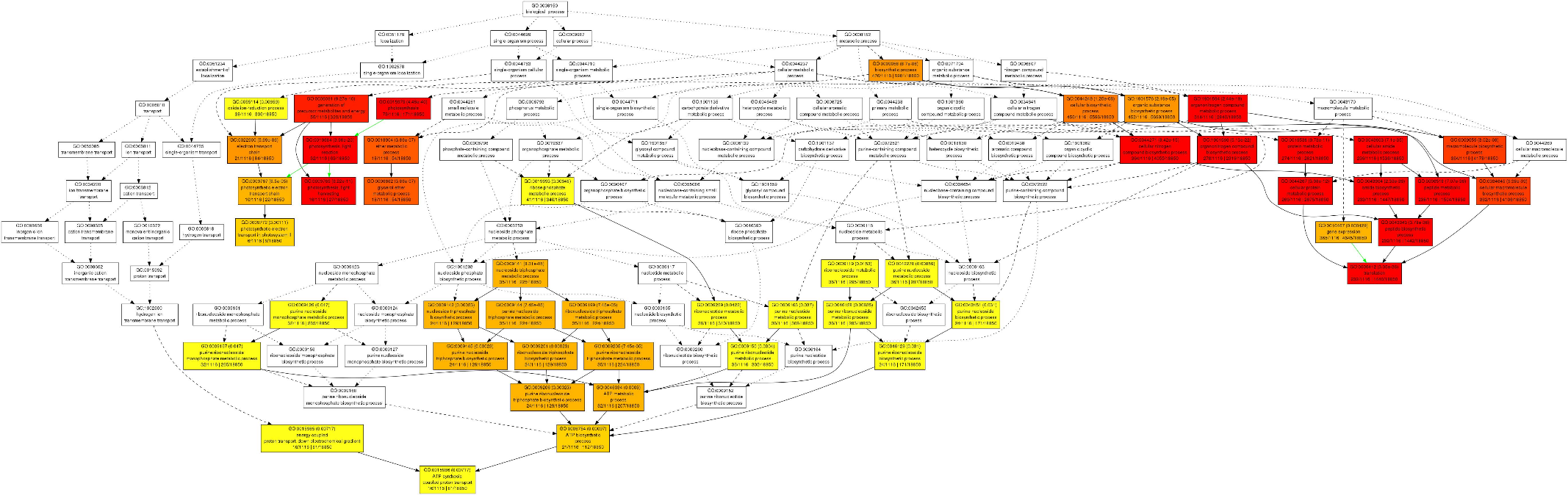


**Supplementary Fig. 7(d): Upregulated GO enriched categories of leaves as compared to rhizomes under biological processes**


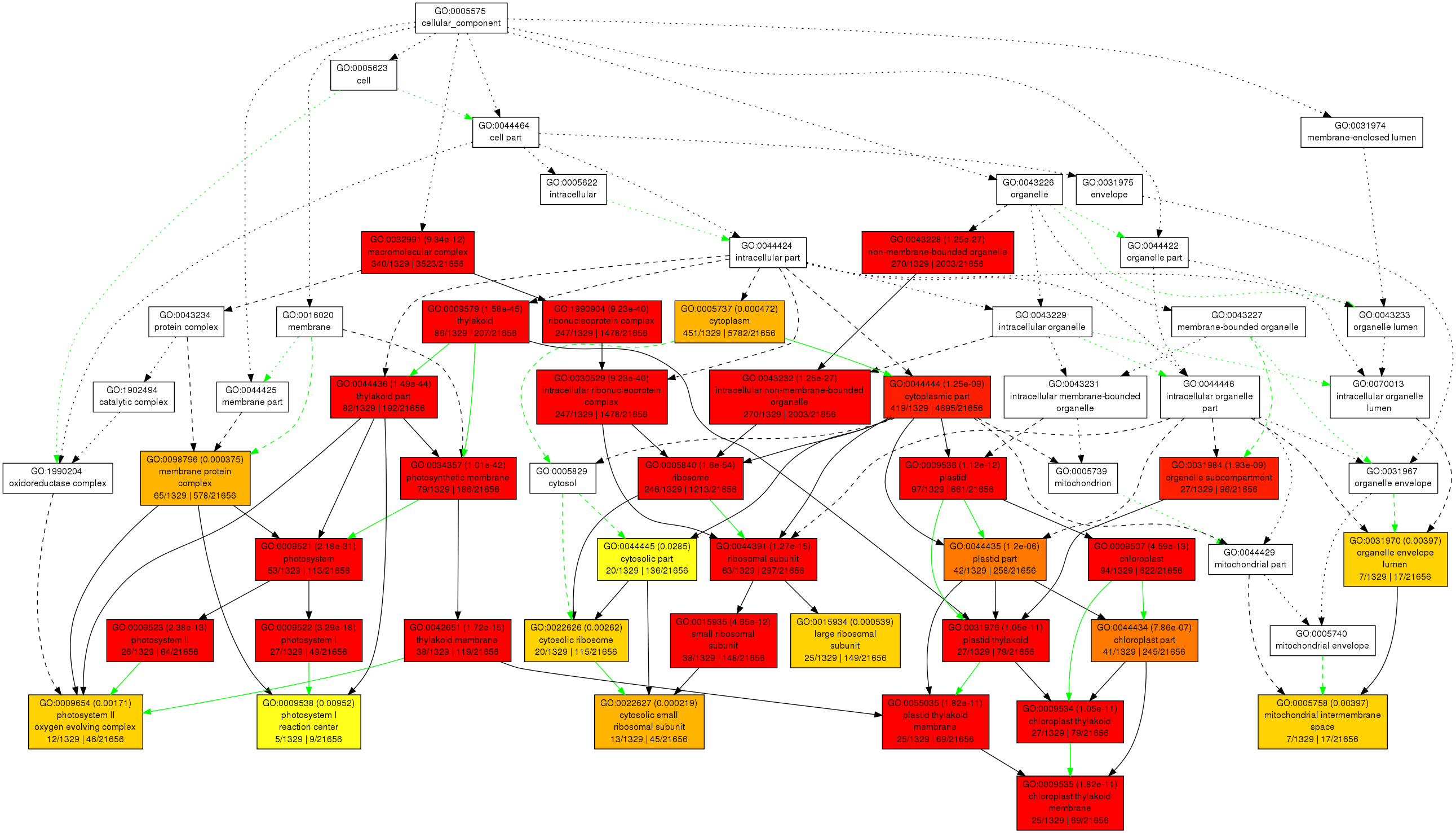


**Supplementary Fig. 7(e): Upregulated GO enriched categories of leaves as compared to rhizomes under cellular components**


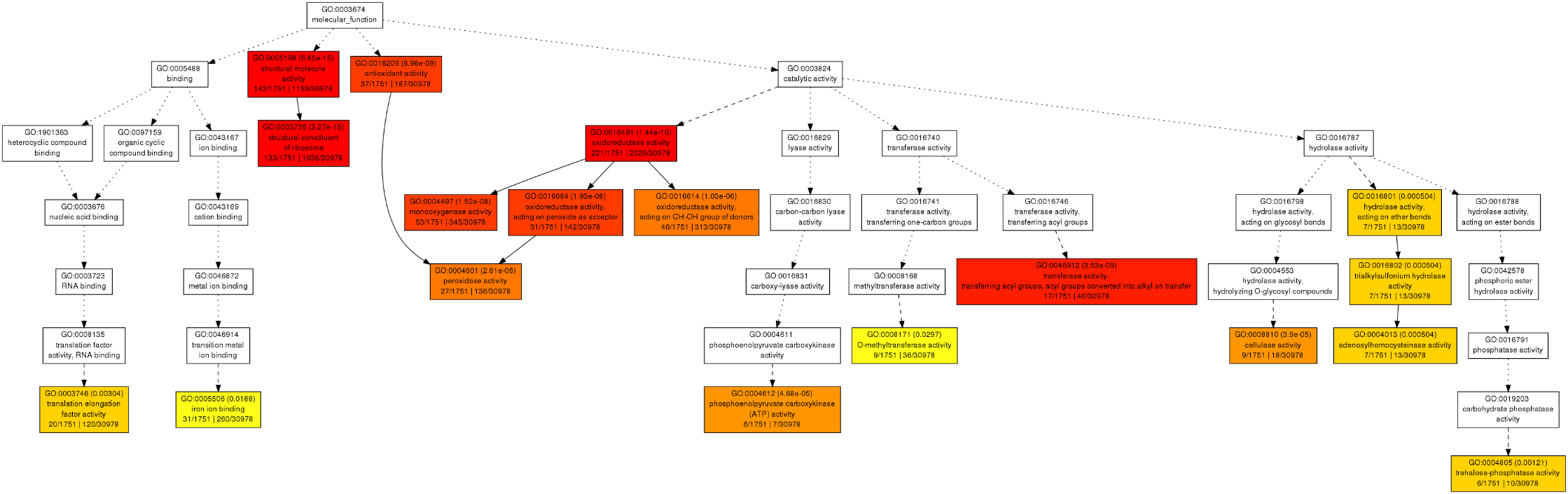


**Supplementary Fig. 7(f): Upregulated GO enriched categories of leaves as compared to rhizomes under molecular functions**

**Supplementary Fig. 7:** GO enrichment analysis in leaves and rhizomes of *N. jatamansi*; Images generated by AgriGO v 2.0 (http:systemsbiology.cau.edu.cn/agriGOv2/c_SEA.php)


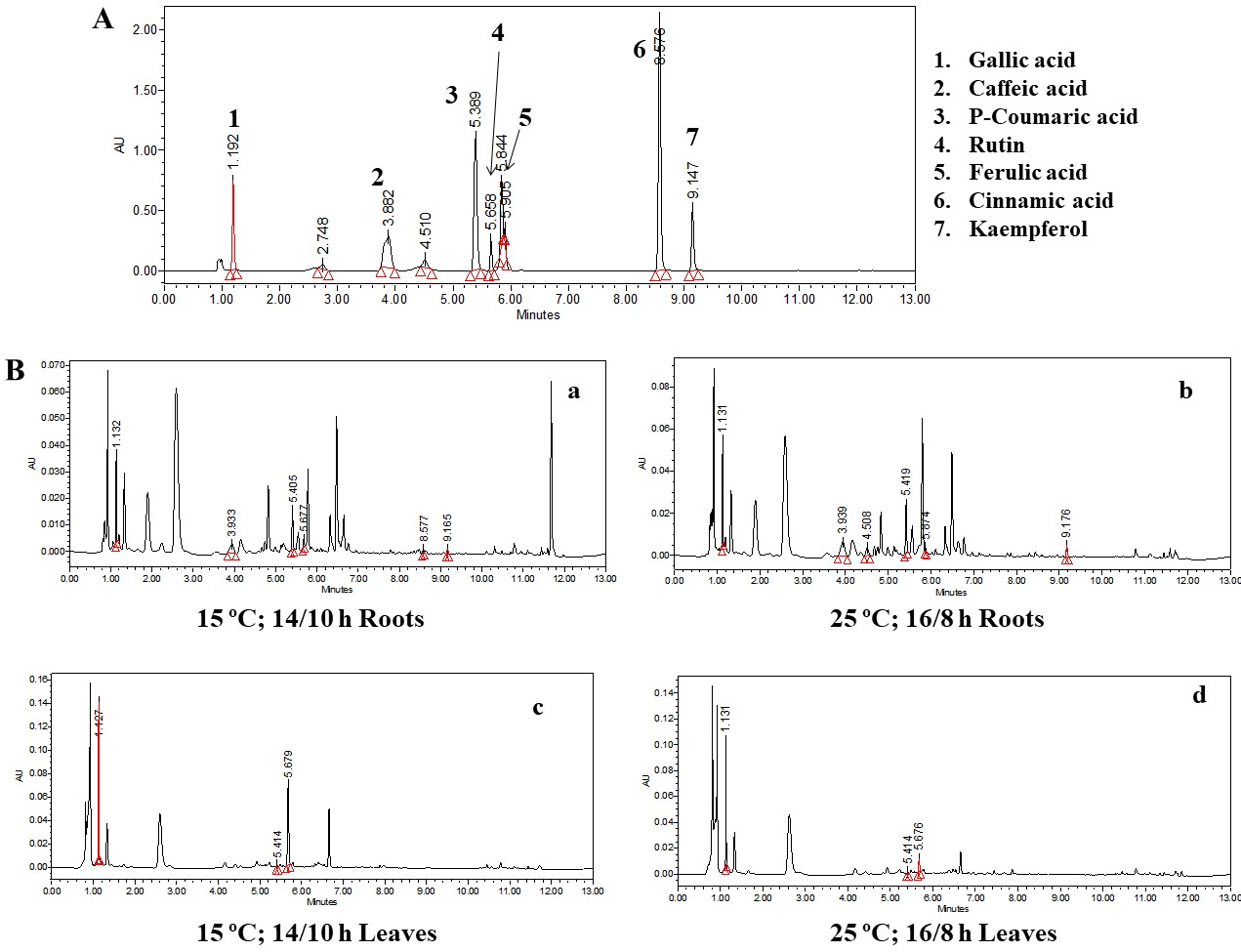


Supplementary Fig. 8: UPLC-MS chromatogram of A) Standard compounds, B) *in vitro* plants of *N. jatamansi* (a) roots at 15 ºC; 14/10 h photoperiod (b) roots at 25 ºC; 16/8 h photoperiod (c) leaves at 15 ºC; 14/10 h photoperiod (b) leaves at 25 ºC; 16/8 h photoperiod

**Legends of Supplementary Figures and Tables:**

**Supplementary Figures:**

Supplementary Fig. 1: Clustering of transcripts of leaves and rhizomes of *N. jatamansi* as per their lengths in base pair

Supplementary Fig. 2: Percent distribution of annotated transcripts of a) leaves and b) rhizomes of *N. jatamansi* based on E-value

Supplementary Fig. 3: Distribution of transcripts of leaves and rhizomes of *N. jatamansi* based on species similarity

Supplementary Fig. 4: Top 20 KEGG pathways in *N. jatamansi* leaves and rhizomes

Supplementary Fig. 5: Different classes of SSRs identified in leaves and rhizomes of *N. jatamansi*

Supplementary Fig. 6: Details of upregulated transcripts from leaves and rhizomes of *N. jatamansi*. LvR_Up represents upregulation in rhizomes as compared to leaves and RvL_Up represents upregulation in leaves as compared to rhizomes

Supplementary Fig. 7: GO enrichment analysis in leaves and rhizomes of *N. jatamansi* (a) Upregulated GO enriched categories of rhizomes as compared to leaves under biological processes, (b) Upregulated GO enriched categories of rhizomes as compared to leaves under cellular components, (c) Upregulated GO enriched categories of rhizomes as compared to leaves under molecular function, (d) Upregulated GO enriched categories of leaves as compared to rhizomes under biological processes, (e) Upregulated GO enriched categories of leaves as compared to rhizomes under cellular components, (f) Upregulated GO enriched categories of leaves as compared to rhizomes under molecular functions. Images generated by AgriGO v 2.0 (http:systemsbiology.cau.edu.cn/agriGOv2/c_SEA.php)

Supplementary Fig. 8: UPLC-MS chromatogram of A) Standard compounds, B) *in vitro* plants of *N. jatamansi* (a) roots at 15 ºC; 14/10 h photoperiod (b) roots at 25 ºC; 16/8 h photoperiod (c) leaves at 15 ºC; 14/10 h photoperiod (b) leaves at 25 ºC; 16/8 h photoperiod

**Supplementary Tables:**

Supplementary Table 1: Statistics of sequencing assembly in *N. jatamansi*

Supplementary Table 2: Distribution of transcripts of leaves and rhizomes of *N. jatamansi* in different KEGG pathways

Supplementary Table 3: Distribution of transcripts of leaves and rhizomes of *N. jatamansi* in different primary and secondary metabolic pathways

Supplementary Table 4: List of differentially expressed genes above log2 fold change + 2 except unannotated DEGs

Supplementary Table 5: Details of transcripts of different transcription factor families identified in leaves and rhizomes of *N. jatamansi*

Supplementary Table 6: Details of Protein–Protein interaction network of DAPs of leaves and rhizomes of *N. jatamansi* identified against known protein-species in Experiment or Text Mining databases

Supplementary Table 7: Details of differentially expressed genes involved in photosynthesis, transport and biosynthesis of precursors of secondary metabolites identified in leaves and rhizomes of *N. jatamansi*; and the list of abbreviations of genes and their full names represented in Fig. 5

Supplementary Table 8: Details of primers used for validation of genes identified through transcriptome analysis of leaves and rhizomes of *N. jatamansi*; and analysis of expression patterns of genes involved in plant’s response to light and temperature
